# 4-[Tris(1*H*-pyrazol-1-yl)meth­yl]phenol

**DOI:** 10.1107/S1600536811043042

**Published:** 2011-10-29

**Authors:** Xiao-Yan Chen, Xiaoping Yang, Bradley J. Holliday

**Affiliations:** a Department of Chemistry and Biochemistry, University of Texas at Austin, 1 University Station, A5300, Austin, TX 78712, USA

## Abstract

The title compound, C_16_H_14_N_6_O, was prepared by the condensation of 4-(trifluoro­meth­yl)phenol and sodium pyrazol-1-ide in a yield of 58%. The dihedral angles formed by the planes of the pyrazole rings are 50.7 (2), 71.2 (3) and 95.8 (2)°. The mol­ecules are associated into dimers by pairs of inter­molecular O—H⋯N hydrogen bonds involving the hy­droxy groups and pyrazole N atoms. In addition, π–π stacking between the phenol rings of these inversion-related dimers is observed, with a ring centroid-to-centroid distance of 3.9247 (10) Å.

## Related literature

For the preparation and coordination chemistry of tris(pyrazol­yl)borates and tris­(pyrazol­yl)methanes, see: Trofimenko (1966 ▶, 1970 ▶, 1999 ▶); Pettinari & Pettinari (2005 ▶); Reger *et al.* (2000 ▶). For the chemistry of tris­(pyrazol­yl)methane derivatives, see: Humphrey *et al.* (1999 ▶). For similar structures, see: Liddle & Gardinier (2007 ▶).
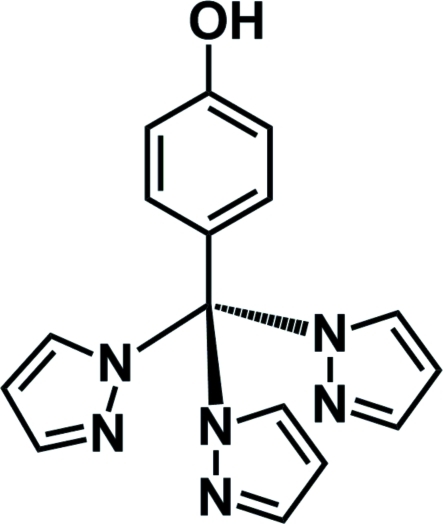


## Experimental

### 

#### Crystal data

C_16_H_14_N_6_O
*M* *_r_* = 306.33
Triclinic, 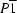

*a* = 8.5065 (17) Å
*b* = 8.6829 (17) Å
*c* = 10.815 (2) Å
α = 96.97 (3)°
β = 91.51 (2)°
γ = 109.40 (3)°
*V* = 746.0 (3) Å^3^
*Z* = 2
Mo *K*α radiation
μ = 0.09 mm^−1^
*T* = 153 K
0.30 × 0.28 × 0.20 mm

#### Data collection

Nonius KappaCCD diffractometer
Absorption correction: Gaussian (*XPREP* in *SHELXTL*; Sheldrick, 2008 ▶) *T*_min_ = 0.973, *T*_max_ = 0.983
4225 measured reflections
2609 independent reflections
1975 reflections with *I* > 2σ(*I*)
*R*_int_ = 0.025

#### Refinement

*R*[*F* ^2^ > 2σ(*F* ^2^)] = 0.043
*wR*(*F* ^2^) = 0.099
*S* = 1.07
2609 reflections
209 parameters
H-atom parameters constrained
Δρ_max_ = 0.18 e Å^−3^
Δρ_min_ = −0.27 e Å^−3^

### 

Data collection: *COLLECT* (Nonius, 1998 ▶); cell refinement: *SCALEPACK* (Otwinowski & Minor, 1997 ▶); data reduction: *DENZO-SMN* (Otwinowski & Minor, 1997 ▶); program(s) used to solve structure: *SIR97* (Altomare *et al.*, 1999 ▶); program(s) used to refine structure: *XL* in *SHELXTL/PC* (Sheldrick, 2008 ▶); molecular graphics: *ORTEP-3* (Farrugia, 1997 ▶) and *POV-RAY* (Persistence of Vision Team, 2004 ▶); software used to prepare material for publication: *SHELXL97* (Sheldrick, 2008 ▶) and *WinGX* (Farrugia, 1999 ▶).

## Supplementary Material

Crystal structure: contains datablock(s) I, global. DOI: 10.1107/S1600536811043042/pk2340sup1.cif

Structure factors: contains datablock(s) I. DOI: 10.1107/S1600536811043042/pk2340Isup2.hkl

Supplementary material file. DOI: 10.1107/S1600536811043042/pk2340Isup3.cml

Additional supplementary materials:  crystallographic information; 3D view; checkCIF report

## Figures and Tables

**Table 1:** Hydrogen-bond geometry (Å, °)

*D*—H⋯*A*	*D*—H	H⋯*A*	*D*⋯*A*	*D*—H⋯*A*
O1—H1*A*⋯N4^i^	0.82	2.02	2.836 (2)	173

Symmetry code: (i) 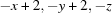.

**Fig. 1.**
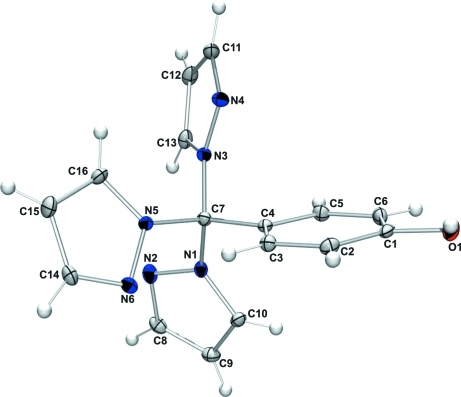
Ellipsoid plot of C16H14N6O showing selected atoms at 30% probability level.

**Fig. 2.**
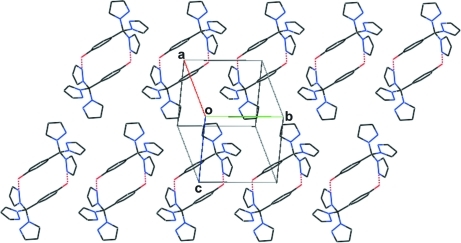
A packing diagram of C16H14N6O.

## supplementary crystallographic information

### Comment

Since seminal reports on tris(pyrazolyl)borates and tris(pyrazolyl)methanes (Trofimenko, 1966, 1970), variations on these ligands have been widely studied. Currently, there are great research efforts to study ligands of this type that are functionalized at the back boron or methine positions. The synthesis and coordination chemistry of aryltris(pyrazolyl)borates have also been well investigated, which has allowed significant advances in iron(II) spin-crossover chemistry. However, the chemistry of analogous tris(pyrazolyl)methane derivatives is almost unknown. Herein, we demonstrate the preparation of a new tris(pyrazolyl)methane derivative, 4-[tris(1*H*-pyrazol-1-yl)methyl]phenol, using a one-pot synthesis method with 4-(trifluoromethyl)phenol and freshly prepared sodium pyrazol-1-ide to give the desired product in good yield.

The solid state structure of 4-[tris(1*H*-pyrazol-1-yl)methyl]phenol can be seen in Fig. 1. There are two molecules in the unit cell. The molecules are associated into dimers by pairs of intermolecular O—H···N hydrogen bonds involving the hydroxyl groups and pyrazole N atoms. In addition, π–π stacking between the phenol rings of these inversion-related (-x + 2, -*y* + 2, -*z*) dimers is observed with a ring centroid-to-centroid distance of 3.9247 (10) Å.

### Experimental

The title compound was prepared by refluxing 4-(trifluoromethyl)phenol (0.162 g, 1.0 mmol) and freshly prepared sodium pyrazol-1-ide (0.361 g, 4.0 mmol) in tetrahydrofuran (20 ml) for 12 h under a nitrogen atomsphere. The desired product was purified by column chromatography on silica gel using CH_2_Cl_2_ as the eluent with a yield of 58%. Single crystals suitable for X-ray diffraction were obtained *via* slow evaporation from a methanol solution.

### Refinement

All H atoms were positioned geometrically and refined using a riding model, with C—H = 0.95 Å, and O—H = 0.82 Å. *U*_iso_(H) values were set to 1.2*U*_eq_(C) or 1.5*U*_eq_(O).

### Crystal data

**Table:**

C_16_H_14_N_6_O	*Z* = 2
*M**_r_* = 306.33	*F*(000) = 320
Triclinic, *P*1	*D*_x_ = 1.364 Mg m^−^^3^
Hall symbol: -P 1	Melting point: 443 K
*a* = 8.5065 (17) Å	Mo *K*α radiation, λ = 0.71073 Å
*b* = 8.6829 (17) Å	Cell parameters from 2208 reflections
*c* = 10.815 (2) Å	θ = 2.9–27.5°
α = 96.97 (3)°	µ = 0.09 mm^−^^1^
β = 91.51 (2)°	*T* = 153 K
γ = 109.40 (3)°	Block, colourless
*V* = 746.0 (3) Å^3^	0.30 × 0.28 × 0.20 mm

### Data collection

**Table:**

Nonius KappaCCD diffractometer	2609 independent reflections
Radiation source: fine-focus sealed tube	1975 reflections with *I* > 2σ(*I*)
graphite	*R*_int_ = 0.025
ω scans	θ_max_ = 25.0°, θ_min_ = 2.9°
Absorption correction: gaussian (*XPREP* in *SHELXTL*; Sheldrick, 2008)	*h* = −10→10
*T*_min_ = 0.973, *T*_max_ = 0.983	*k* = −10→10
4225 measured reflections	*l* = −12→11

### Refinement

**Table:**

Refinement on *F*^2^	Primary atom site location: structure-invariant direct methods
Least-squares matrix: full	Secondary atom site location: difference Fourier map
*R*[*F*^2^ > 2σ(*F*^2^)] = 0.043	Hydrogen site location: inferred from neighbouring sites
*wR*(*F*^2^) = 0.099	H-atom parameters constrained
*S* = 1.07	*w* = 1/[σ^2^(*F*_o_^2^) + (0.038*P*)^2^ + 0.1787*P*] where *P* = (*F*_o_^2^ + 2*F*_c_^2^)/3
2609 reflections	(Δ/σ)_max_ < 0.001
209 parameters	Δρ_max_ = 0.18 e Å^−^^3^
0 restraints	Δρ_min_ = −0.27 e Å^−^^3^

### Special details

**Table:**

Geometry. All e.s.d.'s (except the e.s.d. in the dihedral angle between two l.s. planes) are estimated using the full covariance matrix. The cell e.s.d.'s are taken into account individually in the estimation of e.s.d.'s in distances, angles and torsion angles; correlations between e.s.d.'s in cell parameters are only used when they are defined by crystal symmetry. An approximate (isotropic) treatment of cell e.s.d.'s is used for estimating e.s.d.'s involving l.s. planes.
Refinement. Refinement of *F*^2^ against ALL reflections. The weighted *R*-factor *wR* and goodness of fit *S* are based on *F*^2^, conventional *R*-factors *R* are based on *F*, with *F* set to zero for negative *F*^2^. The threshold expression of *F*^2^ > σ(*F*^2^) is used only for calculating *R*-factors(gt) *etc*. and is not relevant to the choice of reflections for refinement. *R*-factors based on *F*^2^ are statistically about twice as large as those based on *F*, and *R*-factors based on ALL data will be even larger.

### Fractional atomic coordinates and isotropic or equivalent isotropic displacement parameters (Å^2^)

**Table:**

	*x*	*y*	*z*	*U*_iso_*/*U*_eq_	
O1	1.00800 (16)	1.32161 (15)	0.03852 (13)	0.0266 (3)	
H1A	0.9790	1.2970	−0.0362	0.054 (8)*	
N1	0.74972 (17)	0.78542 (18)	0.40566 (13)	0.0180 (4)	
N2	0.69159 (19)	0.65739 (19)	0.47332 (15)	0.0244 (4)	
N3	0.98104 (17)	0.72693 (18)	0.31464 (14)	0.0181 (4)	
N4	1.08607 (18)	0.73336 (19)	0.22092 (15)	0.0237 (4)	
N5	0.70790 (17)	0.60541 (17)	0.21695 (14)	0.0173 (4)	
N6	0.54566 (18)	0.59411 (18)	0.19620 (14)	0.0216 (4)	
C1	0.9528 (2)	1.1846 (2)	0.09625 (17)	0.0197 (4)	
C2	0.8251 (2)	1.0424 (2)	0.04486 (17)	0.0213 (4)	
H2A	0.7695	1.0394	−0.0311	0.026*	
C3	0.7802 (2)	0.9051 (2)	0.10623 (16)	0.0191 (4)	
H3A	0.6944	0.8103	0.0711	0.023*	
C4	0.8613 (2)	0.9069 (2)	0.21937 (16)	0.0165 (4)	
C5	0.9873 (2)	1.0523 (2)	0.27129 (17)	0.0206 (4)	
H5A	1.0427	1.0560	0.3475	0.025*	
C6	1.0305 (2)	1.1896 (2)	0.21160 (17)	0.0213 (4)	
H6A	1.1121	1.2863	0.2487	0.026*	
C7	0.8240 (2)	0.7575 (2)	0.28833 (16)	0.0173 (4)	
C8	0.6124 (2)	0.7133 (2)	0.56240 (18)	0.0250 (5)	
H8A	0.5599	0.6528	0.6241	0.030*	
C9	0.6169 (2)	0.8729 (2)	0.55301 (18)	0.0263 (5)	
H9A	0.5710	0.9371	0.6055	0.032*	
C10	0.7026 (2)	0.9154 (2)	0.45071 (17)	0.0208 (4)	
H10A	0.7246	1.0141	0.4180	0.025*	
C11	1.2086 (2)	0.6894 (2)	0.2675 (2)	0.0268 (5)	
H11A	1.2999	0.6832	0.2244	0.032*	
C12	1.1831 (2)	0.6538 (2)	0.3889 (2)	0.0281 (5)	
H12A	1.2517	0.6210	0.4405	0.034*	
C13	1.0367 (2)	0.6771 (2)	0.41634 (18)	0.0225 (5)	
H13A	0.9846	0.6617	0.4906	0.027*	
C14	0.4724 (2)	0.4422 (2)	0.13858 (17)	0.0220 (4)	
H14A	0.3599	0.3985	0.1111	0.026*	
C15	0.5835 (2)	0.3552 (2)	0.12394 (19)	0.0284 (5)	
H15A	0.5602	0.2468	0.0870	0.034*	
C16	0.7341 (2)	0.4636 (2)	0.17562 (18)	0.0238 (5)	
H16A	0.8347	0.4434	0.1811	0.029*	

### Atomic displacement parameters (Å^2^)

**Table:**

	*U*^11^	*U*^22^	*U*^33^	*U*^12^	*U*^13^	*U*^23^
O1	0.0374 (8)	0.0219 (8)	0.0236 (9)	0.0116 (6)	0.0085 (6)	0.0092 (6)
N1	0.0197 (8)	0.0170 (8)	0.0171 (8)	0.0049 (7)	0.0029 (7)	0.0049 (7)
N2	0.0284 (9)	0.0226 (9)	0.0218 (9)	0.0056 (7)	0.0055 (7)	0.0093 (7)
N3	0.0155 (8)	0.0185 (9)	0.0209 (9)	0.0065 (6)	0.0013 (7)	0.0034 (7)
N4	0.0187 (8)	0.0256 (9)	0.0297 (10)	0.0101 (7)	0.0072 (7)	0.0066 (8)
N5	0.0149 (8)	0.0167 (9)	0.0207 (9)	0.0063 (6)	0.0007 (6)	0.0017 (7)
N6	0.0152 (8)	0.0231 (9)	0.0255 (9)	0.0057 (7)	0.0005 (7)	0.0023 (7)
C1	0.0231 (10)	0.0180 (11)	0.0234 (11)	0.0126 (8)	0.0098 (8)	0.0056 (8)
C2	0.0241 (10)	0.0261 (11)	0.0178 (10)	0.0133 (9)	0.0009 (8)	0.0044 (9)
C3	0.0184 (10)	0.0202 (11)	0.0183 (10)	0.0067 (8)	0.0003 (8)	0.0001 (8)
C4	0.0156 (9)	0.0167 (10)	0.0189 (10)	0.0077 (8)	0.0041 (8)	0.0025 (8)
C5	0.0181 (10)	0.0235 (11)	0.0199 (11)	0.0070 (8)	−0.0014 (8)	0.0020 (8)
C6	0.0210 (10)	0.0167 (10)	0.0235 (11)	0.0032 (8)	0.0030 (8)	0.0011 (8)
C7	0.0145 (9)	0.0186 (10)	0.0188 (10)	0.0061 (8)	0.0008 (8)	0.0017 (8)
C8	0.0210 (10)	0.0329 (12)	0.0185 (11)	0.0049 (9)	0.0037 (8)	0.0048 (9)
C9	0.0228 (11)	0.0321 (12)	0.0248 (11)	0.0120 (9)	0.0039 (9)	−0.0010 (9)
C10	0.0206 (10)	0.0201 (10)	0.0241 (11)	0.0106 (8)	−0.0002 (8)	0.0013 (8)
C11	0.0181 (10)	0.0227 (11)	0.0415 (14)	0.0090 (9)	0.0027 (9)	0.0056 (10)
C12	0.0228 (11)	0.0230 (11)	0.0400 (14)	0.0101 (9)	−0.0097 (9)	0.0057 (10)
C13	0.0255 (11)	0.0191 (11)	0.0223 (11)	0.0066 (8)	−0.0046 (8)	0.0046 (8)
C14	0.0190 (10)	0.0220 (11)	0.0199 (11)	0.0005 (8)	0.0004 (8)	0.0023 (8)
C15	0.0305 (12)	0.0193 (11)	0.0309 (12)	0.0053 (9)	0.0004 (9)	−0.0042 (9)
C16	0.0253 (11)	0.0198 (11)	0.0297 (12)	0.0128 (9)	0.0023 (9)	0.0012 (9)

### Geometric parameters (Å, °)

**Table:**

O1—C1	1.360 (2)	C4—C5	1.399 (3)
O1—H1A	0.8200	C4—C7	1.521 (3)
N1—C10	1.360 (2)	C5—C6	1.371 (3)
N1—N2	1.366 (2)	C5—H5A	0.9300
N1—C7	1.462 (2)	C6—H6A	0.9300
N2—C8	1.325 (2)	C8—C9	1.390 (3)
N3—C13	1.358 (2)	C8—H8A	0.9300
N3—N4	1.363 (2)	C9—C10	1.362 (3)
N3—C7	1.473 (2)	C9—H9A	0.9300
N4—C11	1.330 (2)	C10—H10A	0.9300
N5—C16	1.349 (2)	C11—C12	1.391 (3)
N5—N6	1.361 (2)	C11—H11A	0.9300
N5—C7	1.470 (2)	C12—C13	1.361 (3)
N6—C14	1.323 (2)	C12—H12A	0.9300
C1—C6	1.385 (3)	C13—H13A	0.9300
C1—C2	1.387 (3)	C14—C15	1.393 (3)
C2—C3	1.382 (3)	C14—H14A	0.9300
C2—H2A	0.9300	C15—C16	1.369 (3)
C3—C4	1.385 (2)	C15—H15A	0.9300
C3—H3A	0.9300	C16—H16A	0.9300
			
C1—O1—H1A	109.5	N1—C7—N3	109.47 (14)
C10—N1—N2	111.56 (15)	N5—C7—N3	107.01 (14)
C10—N1—C7	128.86 (15)	N1—C7—C4	110.73 (14)
N2—N1—C7	118.61 (14)	N5—C7—C4	113.65 (14)
C8—N2—N1	104.13 (15)	N3—C7—C4	108.88 (14)
C13—N3—N4	111.24 (14)	N2—C8—C9	112.15 (18)
C13—N3—C7	130.58 (16)	N2—C8—H8A	123.9
N4—N3—C7	117.89 (14)	C9—C8—H8A	123.9
C11—N4—N3	104.65 (15)	C10—C9—C8	105.46 (18)
C16—N5—N6	112.33 (15)	C10—C9—H9A	127.3
C16—N5—C7	129.11 (15)	C8—C9—H9A	127.3
N6—N5—C7	118.30 (14)	N1—C10—C9	106.65 (17)
C14—N6—N5	103.95 (14)	N1—C10—H10A	126.7
O1—C1—C6	117.37 (17)	C9—C10—H10A	126.7
O1—C1—C2	123.26 (17)	N4—C11—C12	111.55 (17)
C6—C1—C2	119.36 (17)	N4—C11—H11A	124.2
C3—C2—C1	120.19 (17)	C12—C11—H11A	124.2
C3—C2—H2A	119.9	C13—C12—C11	105.54 (17)
C1—C2—H2A	119.9	C13—C12—H12A	127.2
C2—C3—C4	120.83 (17)	C11—C12—H12A	127.2
C2—C3—H3A	119.6	N3—C13—C12	107.00 (18)
C4—C3—H3A	119.6	N3—C13—H13A	126.5
C3—C4—C5	118.31 (17)	C12—C13—H13A	126.5
C3—C4—C7	123.42 (16)	N6—C14—C15	112.14 (17)
C5—C4—C7	118.25 (16)	N6—C14—H14A	123.9
C6—C5—C4	121.01 (17)	C15—C14—H14A	123.9
C6—C5—H5A	119.5	C16—C15—C14	105.15 (17)
C4—C5—H5A	119.5	C16—C15—H15A	127.4
C5—C6—C1	120.22 (17)	C14—C15—H15A	127.4
C5—C6—H6A	119.9	N5—C16—C15	106.41 (17)
C1—C6—H6A	119.9	N5—C16—H16A	126.8
N1—C7—N5	106.98 (14)	C15—C16—H16A	126.8

### Hydrogen-bond geometry (Å, °)

**Table:**

*D*—H···*A*	*D*—H	H···*A*	*D*···*A*	*D*—H···*A*
O1—H1A···N4^i^	0.82	2.02	2.836 (2)	173.

Symmetry codes: (i) −*x*+2, −*y*+2, −*z*.
